
JOURNAL INFORMATION
==============================
NLM Title Abbreviation: Behav Neurol
Journal ID: Behav Neurol
NLM Journal ID: 8914585
Journal ID: BN

Behavioural Neurology

ISSN: 0953-4180
EISSN: 1875-8584
Publisher: Wiley

ARTICLE INFORMATION
==============================
PMCID: PMC5471543
PMID: 28670099
DOI: 10.1155/2006/298756
Article ID: 298756
Article version: 1
Subjects: Research Article

Neuropharmacologic Approaches to Cognitive Rehabilitation

Klein Reva
McNamara Patrick
Albert Martin L. *
Harold Goodglass Aphasia Research Center Department of Neurology Boston University Medical School and the Medical Research Service VA Boston Healthcare System USA
*Martin L. Albert: malbert@bu.edu
Print publication date: 2006
Electronic publication date: 2006 May 11
Volume: 17
Issue: 1
First page: 1
Last page: 3
Received 2006 Apr 26; Accepted 2006 Apr 26
Copyright: Copyright © 2006 Hindawi Publishing Corporation and the authors.
Copyright year: 2006
License: This is an open access article distributed under the Creative Commons Attribution License, which permits unrestricted use, distribution, and reproduction in any medium, provided the original work is properly cited.
License URL: https://creativecommons.org/licenses/by/3.0/

==============================
Behavioural Neurology 17 (2006) 1–3
IOS Press

### Neuropharmacologic approaches to cognitive rehabilitation

Reva Klein, Patrick McNamara and Martin L. Albert*
*Harold Goodglass Aphasia Research Center, Department of Neurology, Boston University Medical School and the Medical Research Service, VA Boston Healthcare System, USA*

Neuropharmacologic rehabilitation of cognitive deficits is a field waiting to be created. NIH, with its Roadmap Initiative for Translational Research, is pushing hard, but clinicians and scientists, for the most part, have yet to accept the challenge. The goal of this Special Issue is to document the current state of the art. As readers will note, most contributors conclude the field is just now taking its tentative first steps toward self-definition.

Our aim in this Special Issue is to present a survey of the latest developments in pharmacologic treatment of cognitive deficit in selected clinical disorders, including the dementias, Parkinson's disease (PD), multiple sclerosis (MS), epilepsy, vascular disorders and traumatic brain injury (TBI). Our primary interest was to consider not only treatment of acute or subacute disease, but also approaches to prevention and rehabilitation. Two principal conclusions emerge from these detailed reviews: 1) cognitive disorders associated with each of these neurologic conditions can be severe and disabling; 2) effective pharmacologic intervention for some of these disabling cognitive symptoms are now emerging, but are understudied.

The first three papers discuss disorders (dementias, vascular disorders and TBI) that share a long tradition of research into cognitive deficits. Thus, studies of pharmacologic management of cognitive disturbances in these disorders are considerably more advanced than those for disorders discussed in the last three papers (PD, MS and epilepsy). Development of effective treatments for cognitive disorders in these last three disorders had to await recognition and typing of the variety, extent and severity of cognitive deficit. This evaluative and classificatory work has largely been accomplished only in the last 15 or so years.

In their review of the dementias, Ringman and Cummings note that the cholinesterase inhibitors donepezil, rivastigmine, and galantamine have demonstrated efficacy in improving cognition and global status in mild to moderate Alzheimer's disease. Donepezil and galantamine have also been shown to be mildly effective in dementia due to cerebral ischemia. Only memantine has demonstrated some efficacy in moderate to severe AD. The benefits from these drugs, however, are limited and their long-term effectiveness has not been well-studied. Unfortunately, there are currently no proven treatments directed specifically at the pathology of frontotemporal dementia and no currently proven therapy for the prevention of dementia.

In their paper on cerebrovascular disorders, Romero and his colleagues review recent research on basic mechanisms of neuronal injury in the ischemic penumbra, focusing on new opportunities to promote recovery after injury or to protect against cellular damage in the first place. This basic research has revealed one of the most spectacular finds in the recent history of the science of neurorehabilitation. The adult human brain in the areas of the subventricular zone and the hippocampal dentate gyrus (deep to the temporal lobe) contains populations of undifferentiated

*Corresponding author: Martin L. Albert, M.D., Ph.D., Aphasia Research Center (12A), VA Boston Healthcare System, 150 So. Huntington Avenue, Boston, MA 02130, USA. Tel.: +1 857 364 4775; Fax: +1 617 739 8926; E-mail: malbert@bu.edu.

ISSN 0953-4180/06/$17.00 © 2006 – IOS Press and the authors. All rights reserved progenitor cells that can, under the right conditions, proliferate and differentiate (after migrating to the olfactory bulb) into mature brain cells. Following ischemic injury some of these cells may migrate to the periphery of the infarct as well and thus could theoretically facilitate recovery of cellular function. Growth factors can promote differentiation of progenitor cells into mature neurons and represent a promising new area in the treatment of stroke.

In their paper on pharmacotherapy of posttraumatic cognitive impairments, Arciniegas and Silver note that augmentation of posttraumatic cerebral catecholaminergic and cholinergic function has demonstrated some efficacy in improving cognition in patients with TBI. When the posttraumatic impairments are in the areas of arousal, speed of processing, and attention, agents that enhance catecholaminergic transmission (such as methylphenidate, dextroamphetamine, amantadine, or bromocriptine) are more effective than cholinergic agents. In severe cases, modafanil, carbidopa/L-dopa, or other non-standard agents with stimulating properties such as modafinil, protriptytline, or lamotrigine have sometimes been effective. When the predominant posttraumatic impairment is in the domain of memory, cholinergic agents (such as cholinesterase inhibitors) are recommended. The authors are careful to point out that pharmacotherapy is only one of several possible interventions for posttraumatic cognitive impairments, and is at present best regarded as an adjunct to nonpharmacologic therapies for such problems.

In their paper on treatment of cognitive deficits in PD, McNamara and Durso note that many patients with PD experience significant cognitive impairment in the realm of executive function, even early in the course of the disease. These mental impairments are only partially responsive to levodopa treatment and are often as disabling as the motor impairment, particularly in the mid and late stages of the disease. Although there has been only a handful of properly controlled clinical trials of intervention targeted at amelioration of mental dysfunction in PD, catecholaminergic and cholinergic agents have demonstrated efficacy, though not as yet over the long-term.

In their paper on MS, Pierson and Griffith note that cognitive impairments in multiple sclerosis are now recognized to be substantial, with the domains of executive function and speed of processing most severely affected. While it may be intuitive to link effective treatment of fatigue with improvement in cognitive performance, studies have yet to bear that out. Emotional well-being and pharmacologic stabilization of associated affective disorders can have a positive influence on cognitive performance in MS. However, significant deficits may remain even after treatment of depression.

In their paper on epilepsy Mula and Trimble note that it is now acknowledged that disorders of attention and memory are frequent in patients with epilepsy. Sources of these cognitive deficits likely involve some combination of the underlying pathology (e.g. hippocampal sclerosis and/or ictal and interictal neurophysiological disturbances) and antiepileptic drugs themselves. Piracetam and its derivatives, and sabeluzole, have received some support for treatment of these cognitive deficits in epilepsy. The authors highlight the need and promise of focusing on neuroprotection in future studies of epilepsy, and they note that a number of psychotropic agents enhance synaptogenesis and neurogenesis while improving cognition.

Striking themes emerge from these reviews: 1) while dopaminergic and cholinergic agents have the best track records to date (most efficacious in reducing deficit), noradrenergic agents are fast emerging as promising agents for treatment of cognitive deficits in neurologic disorders; 2) serotonergic agents have been assessed and found wanting (though they certainly need more thorough evaluations before they can be dismissed as ineffective in the cognitive realm); 3) treatment of depressive and other mood disorders in these various neurologic disorders often slightly improves cognitive dysfunction, although significant residual dysfunction remains, indicating that cognitive deficits in these disorders are not due primarily to mood problems; 4) pharmacotherapy is typically more effective when combined with cognitive-behavioral interventions; 5) neuroprotective agents may also enhance cognition; these agents should receive more attention; 6) atypical antipsychotics that are effective for treatment of cognitive disorders in schizophrenia may prove useful for treatment of both neuropsychiatric complications and selected cognitive disorders in the neurologic disorder reviewed here; 7) side effect profiles for all of the major agents discussed in these papers vary considerably across disorders, and, thus, need to be catalogued with each new application of the drug; 8) drug interactions and polypharmacy are unresolved problems.

Finally, we propose the establishment of a national database on drugs potentially effective in treating cognitive processes or symptoms in neurologic disorders. Currently the neuropharmacologic literature is organized along traditional medical lines according to disorder. While such an approach to classification is certainly valuable, an alternative approach that targets selected subsystems or subprocesses within a larger cognitive processing module might be useful as well. Such a database could be clinically useful in helping to identify promising new treatments across disorder types. For example, episodic memory lapses are common features of many neurologic conditions and perusal of the reviews in this Special Issue suggest that a small class of cholinesterase inhibitors may be effective in treating these memory lapses regardless of the neurologic diagnostic label.

We thank the authors of the papers in this Special Issue, and the editors for inviting us to put it together. The clinical cognitive neurosciences are poised for significant breakthroughs in treatment of cognitive dysfunction.

### Acknowledgements

Supported in part by NIH (NIDCD and NIA) and VA Medical Research Service.
